# Right Hemicolectomy for Refractory Hematochezia in Cecal Angiodysplasia After Failed Embolization: A Case Report

**DOI:** 10.7759/cureus.109490

**Published:** 2026-05-23

**Authors:** Carlos J Mares Jimenez, Armando Viramontes Perez, Dulce N Montes Herrera, Danahe A Chavez Loya, Islamar B Leyva Hernández

**Affiliations:** 1 General Surgery, Clínica Hospital Instituto de Seguridad y Servicios Sociales de los Trabajadores del Estado (ISSSTE) Ensenada, Ensenada, MEX; 2 Medical Intern, Clínica Hospital Instituto de Seguridad y Servicios Sociales de los Trabajadores del Estado (ISSSTE) Ensenada, Ensenada, MEX

**Keywords:** angiodysplasia, case reports, colectomy, embolization, gastrointestinal hemorrhage, therapeutic

## Abstract

Angiodysplasia is an acquired vascular malformation that accounts for a substantial proportion of lower gastrointestinal bleeding in older adults, most often involving the cecum and ascending colon. Although endoscopic and endovascular therapies are considered first-line treatments, a subset of patients develops refractory bleeding that requires a multimodal approach. The objective of this case report is to highlight the management of early endovascular failure in a well-localized cecal angiodysplasia and to illustrate the role of timely right hemicolectomy in achieving sustained remission.

We describe a 67-year-old Hispanic man, a former school teacher with long-standing hypertension and chronic diclofenac use, who presented with recurrent hematochezia and severe iron-deficiency anemia. Despite multiple endoscopic evaluations and medical therapy with proton pump inhibitors and somatostatin analogs, bleeding persisted. Dynamic computed tomography angiography demonstrated cecal angiodysplasia, and selective transcatheter arterial embolization using distal microparticles was performed. However, early rebleeding occurred within ten days, most likely due to rapid vascular recanalization of the embolized branch, a known limitation of particle embolization in this territory, ultimately necessitating right hemicolectomy, which achieved durable control of overt bleeding at six‑month follow‑up.

This case illustrates the limitations of endovascular therapy in refractory angiodysplasia and underscores the importance of careful vascular assessment when selecting embolic agents, particularly in patients with proangiogenic factors such as chronic nonsteroidal anti-inflammatory drug (NSAID) exposure. We briefly review current evidence on the diagnostic role of dynamic computed tomography angiography and the position of surgery within a multidisciplinary strategy for complex gastrointestinal angiodysplasia. Refractory gastrointestinal angiodysplasia should be approached as a systemic vascular disorder requiring individualized, multimodal management.

Even in the era of advanced endoscopic and endovascular therapies, surgery remains an essential definitive option for selected patients in whom bleeding persists despite optimized minimally invasive interventions. From the patient’s perspective, definitive surgery was perceived as a last resort but an ultimately reassuring solution after months of transfusion-dependent bleeding.

## Introduction

Angiodysplasia is an acquired vascular malformation characterized by dilated, tortuous vessels located primarily in the mucosa and submucosa of the gastrointestinal tract. It is one of the most frequent vascular diseases of the digestive system and a leading cause of recurrent lower gastrointestinal bleeding in older adults, second only to diverticular disease. Classically, angiodysplastic lesions have been reported most often in the cecum and ascending colon; however, the increasing use of capsule endoscopy and device-assisted enteroscopy has revealed that the small bowel is also a common site of involvement [1-2].

The pathophysiology of gastrointestinal angiodysplasia is multifactorial and remains incompletely understood. Proposed mechanisms include chronic venous obstruction and increased wall tension, leading to submucosal congestion and angiogenesis, as well as chronic hypoxia with overexpression of proangiogenic factors such as vascular endothelial growth factor. These mechanisms are frequently associated with comorbid conditions, including aortic stenosis, chronic kidney disease, cirrhosis, and cardiovascular disease, which are recognized risk factors for angiodysplasia and recurrent bleeding [1-2]. Chronic nonsteroidal anti-inflammatory drug (NSAID) use may further exacerbate angiodysplastic bleeding by impairing platelet aggregation and mucosal integrity, amplifying the impact of these hemodynamic and angiogenic abnormalities, and lowering the threshold for clinically overt hemorrhage compared with other causes of lower gastrointestinal bleeding, such as peptic ulcer disease or diverticular bleeding.

In our patient, these pathophysiological mechanisms help explain why a seemingly localized cecal lesion behaved in a refractory manner despite technically successful embolization. The combination of increased wall tension in the right colon, chronic NSAID‑related mucosal injury, and a proangiogenic milieu likely promoted rapid neovascularization and reopening of fragile angiodysplastic channels within the embolized territory. This pattern challenges the common assumption that a focal colonic angiodysplasia identified on angiography is almost always definitively controllable by endovascular means, and suggests that “localized” disease in a high‑risk vascular bed such as the cecum may still require early surgical consideration once recurrent bleeding occurs after an adequate embolization attempt.

Currently, endoscopic therapies and transcatheter arterial embolization constitute first-line approaches for the management of bleeding angiodysplasia, while pharmacologic options such as somatostatin analogs and other antiangiogenic agents are increasingly used in refractory or multifocal disease [2]. Nevertheless, a subset of patients continue to experience recurrent or severe bleeding despite optimized minimally invasive management, and surgery remains the only definitive treatment in these cases [2]. The objective of this case report is to highlight the management of early endovascular failure in a well‑localized cecal angiodysplasia and to illustrate how dynamic computed tomography angiography can guide timely, definitive right hemicolectomy within a multidisciplinary strategy to achieve durable control of overt bleeding.

This case report was prepared following the CAse REports (CARE) Guidelines.

## Case presentation

A 67-year-old Hispanic man, a former school teacher with long-standing hypertension treated with losartan and metoprolol and chronic diclofenac use for bilateral knee osteoarthritis, presented to the emergency department with persistent hematochezia (approximately six episodes per day) and symptoms of anemia, including syncope, asthenia, and adynamia. Initial laboratory tests revealed severe anemia, with a hemoglobin level of 6.7 g/dL (reference range 13.7-17.5 g/dL for adult men) and preserved renal function, with a serum creatinine of 0.9 mg/dL, blood urea nitrogen of 22.4 mg/dL, and an estimated glomerular filtration rate (eGFR) greater than 60 mL/min/1.73 m². Diclofenac was discontinued on admission and was not restarted during follow-up. The patient denied other gastrointestinal or constitutional symptoms.

**Figure 1:**
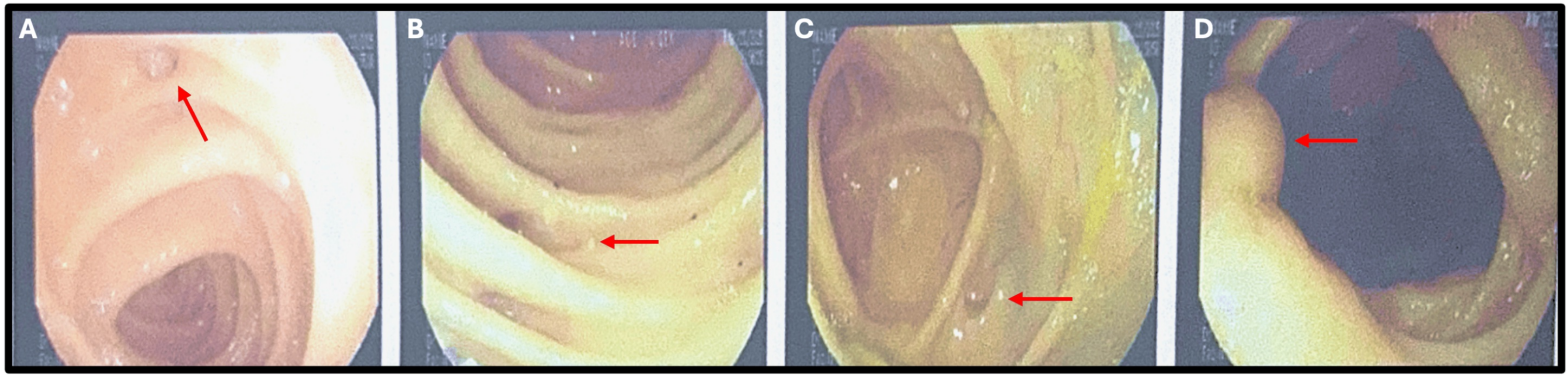
Initial diagnostic dilemma: negative urgent colonoscopy. Images labeled A–D from an urgent colonoscopy performed on admission, showing multiple diverticula predominantly in the ascending colon and cecum and grade II internal hemorrhoidal disease, without active bleeding or stigmata of recent hemorrhage; arrows indicate diverticular orifices.

The patient received fluid resuscitation and red blood cell transfusions (a total of three units of packed red blood cells during the initial hospitalization), followed by an urgent standard colonoscopy to evaluate active lower gastrointestinal bleeding. Colonoscopy showed diverticula predominantly in the ascending colon and cecum, as well as grade II hemorrhoidal disease, but no active bleeding or stigmata of recent hemorrhage (Figure 1).

**Figure 2:**
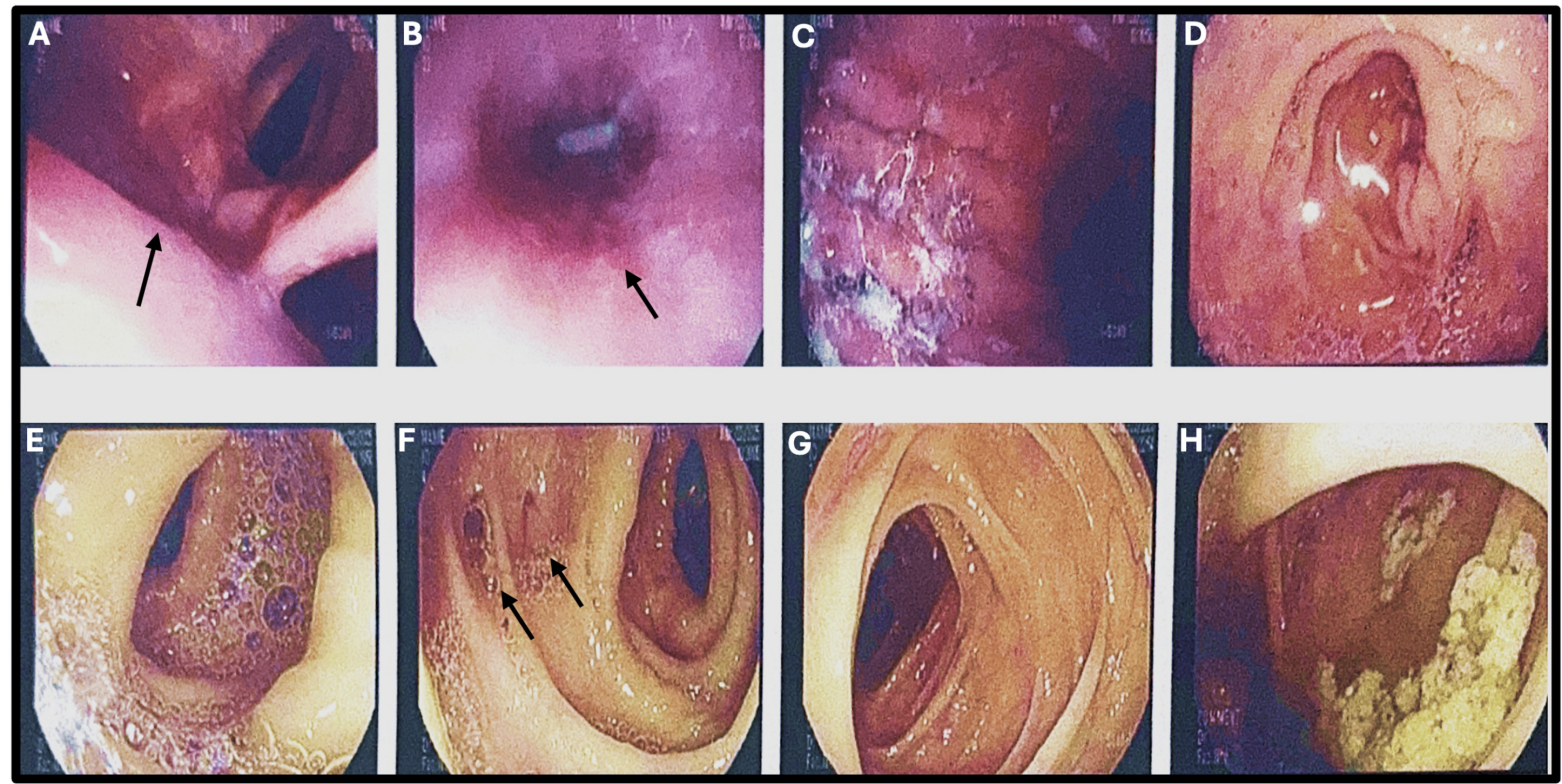
Second endoscopic evaluation. Sequential upper and lower endoscopic images labeled A–H. A and B: Upper endoscopy showing blood remnants in the gastric fundus and superficial erosions without stigmata of active or recent bleeding; arrows indicate areas of mucosal injury. C and D: Additional gastric views demonstrating erosive gastropathy without fresh blood. E and F: Distal small bowel views without a clearly identifiable focal bleeding source. G and H: Colonic views showing residual luminal contents without active bleeding.

Because hematochezia persisted, a second endoscopic evaluation with upper endoscopy and repeat colonoscopy was performed 48 hours later. Upper endoscopy revealed blood remnants in the gastric fundus and superficial erosions without stigmata of active bleeding, whereas colonoscopy again demonstrated multiple diverticula without active hemorrhage (Figure 2).

Given the life‑threatening degree of anemia, these superficial gastric erosions were considered clinically insufficient to account for the magnitude of the hemoglobin drop, and therefore, the diagnostic workup was continued. Conservative management with proton pump inhibitors, short‑acting octreotide, and misoprostol was initiated; octreotide was administered at a dose of 50 µg subcutaneously every 8 hours, and misoprostol at 200 mg orally every 6 hours for 20 days, achieving only partial remission. Bowel movements temporarily normalized (Bristol stool type 4) over 72 hours, and hemoglobin levels remained above 8 g/dL.

**Figure 3:**
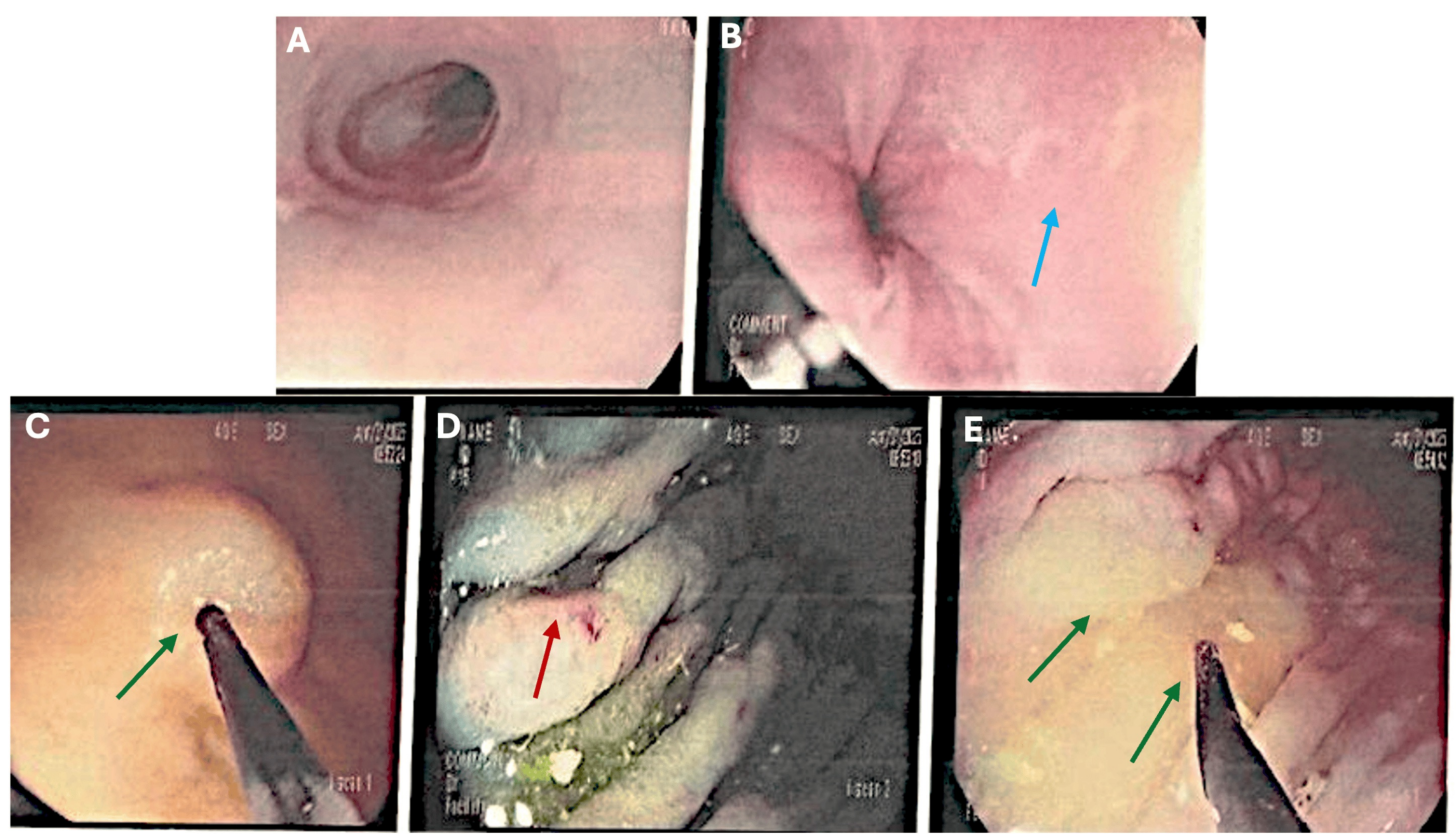
Therapeutic upper endoscopy. Upper endoscopy images labeled A–E showing edema and erosions in the gastric body and fundus without fresh blood. A: Normal-appearing esophageal mucosa. B: Gastric body with mucosal edema and erosions; the blue arrow indicates mucosal edema and erosions. C and E: Gastric erosions treated with dilute epinephrine injection (1:10,000 in normal saline; total volume 5 mL); the green arrows indicate the sites of epinephrine injection. D: Gastric erosions with mucosal friability and heme staining; the red arrow indicates areas of friable mucosa suspicious but not definitive for recent hemorrhage. These lesions were treated endoscopically but were not considered sufficient to explain the severity and persistence of bleeding.

Despite these measures, the patient developed new intermittent episodes of melena and hematochezia, with an abrupt hemoglobin drop to 5.2 g/dL (reference range 13.7-17.5 g/dL for adult men), consistent with life‑threatening anemia, requiring additional transfusions of four units of packed red blood cells. A repeat upper endoscopy showed similar gastric erosions, and endoscopic therapy with dilute epinephrine injection (1:10,000 in normal saline; total volume 5 mL) was performed at the erosion sites (Figure 3).

Although there was a transient improvement, hematochezia recurred shortly thereafter, and no clear source of active bleeding could be identified on conventional endoscopy, making the gastric findings less likely to explain the persistent lower gastrointestinal bleeding. Given that the treated gastric erosions were judged insufficient to account for the severity and persistence of bleeding, a diagnostic workup for obscure gastrointestinal bleeding was initiated, and the differential diagnosis included small bowel angiodysplasia, other causes of obscure small bowel bleeding, and, less likely, intermittent diverticular or hemorrhoidal bleeding.

**Figure 4:**
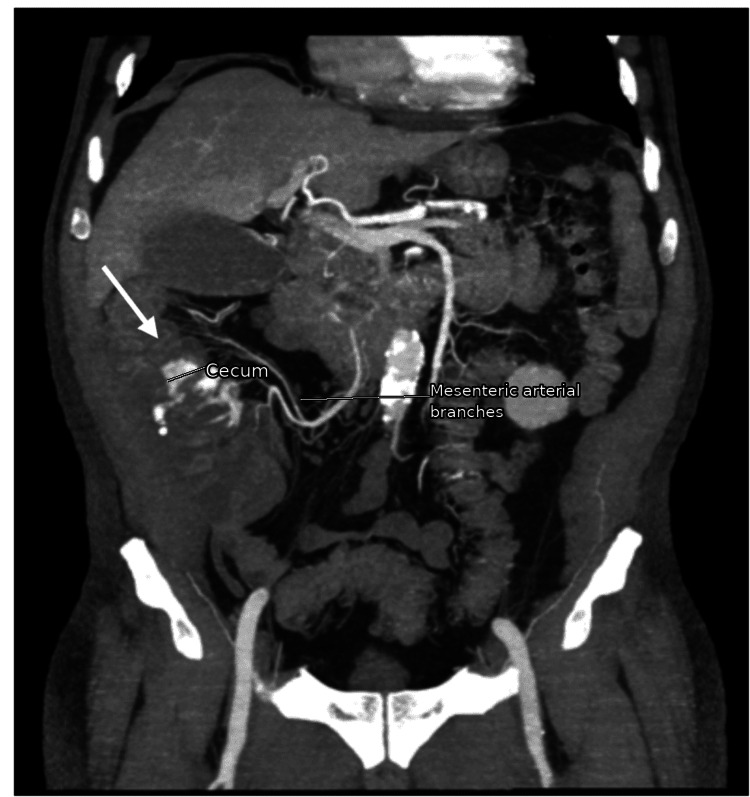
Dynamic CT angiography of the cecum. Coronal arterial-phase computed tomography angiography performed with a lower gastrointestinal bleeding protocol, demonstrating active contrast extravasation in the cecum (white arrow) and cecal vascular ectasia consistent with bleeding from angiodysplasia. The cecum and adjacent mesenteric arterial branches are labeled to illustrate the pathological anatomy and precise location of the bleeding focus.

Dynamic computed tomography angiography (CTA) demonstrated arterial-phase contrast extravasation and vascular ectasia in the cecum, highly suggestive of angiodysplasia (Figure 4). Early venous filling of the draining mesenteric veins was not documented in this study, but the combination of arterial-phase extravasation and cecal vascular ectasia remained highly suggestive of cecal angiodysplasia. CTA was performed with arterial-phase acquisition on a multidetector CT scanner (169 slices in the arterial phase) using a standard lower gastrointestinal bleeding protocol to optimize detection of active contrast extravasation and angiodysplastic vascular tufts, and calcified atherosclerotic changes of the abdominal aorta and its major branches were also noted, without aneurysmal dilatation or focal stenosis.

**Figure 5:**
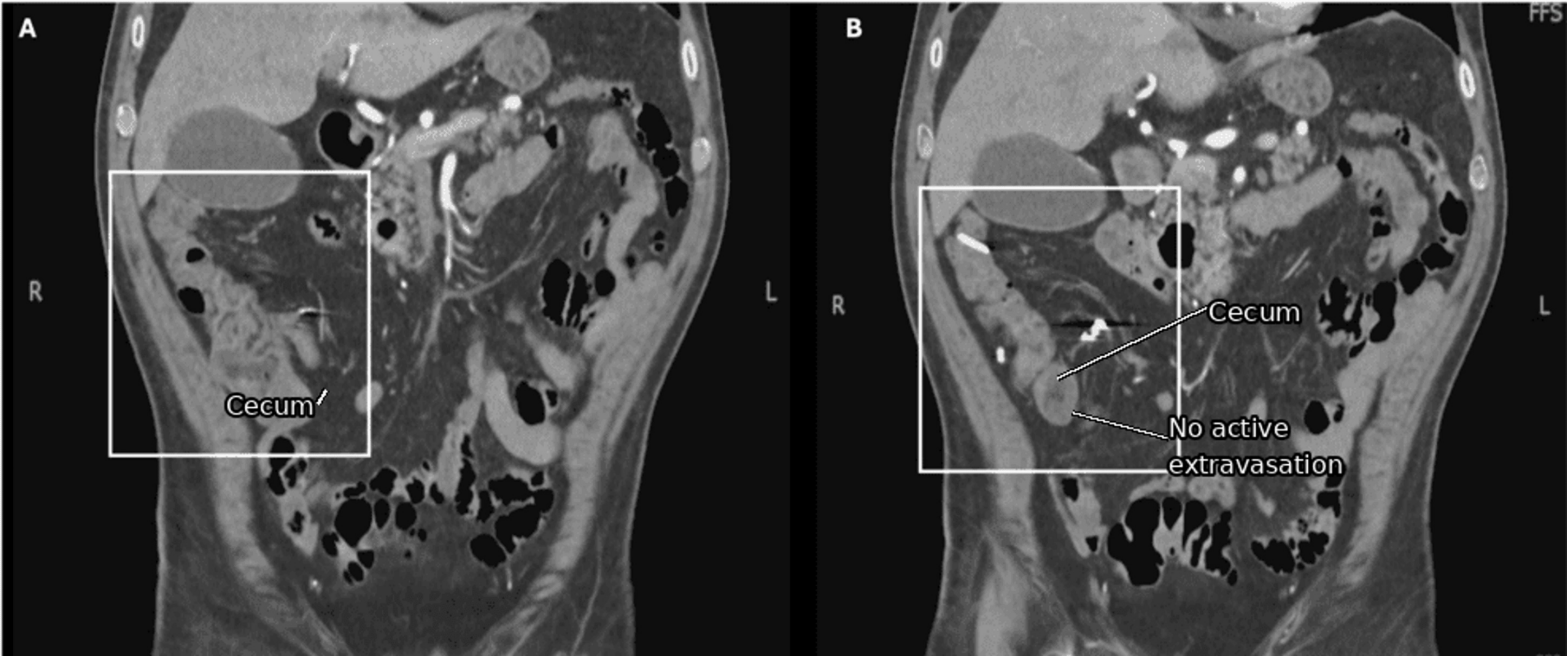
Follow-up CT angiography after embolization of cecal angiodysplasia. Coronal arterial-phase CT angiography (CTA) at the same level as Figure 4. Panel A shows the region of the cecum (white box) without visible active contrast extravasation. Panel B shows the same cecal region labeled “Cecum” and “No active extravasation” to highlight the previous embolization site within the box and to illustrate the diagnostic limitation of a negative post-embolization CTA despite persistent clinical bleeding.

The case was then reviewed with the interventional radiology team, and superselective transcatheter arterial embolization of the cecal lesion was performed via the ileocolic arterial branches using a microcatheter, with distal embolization using small‑caliber microparticles that achieved angiographic cessation of contrast extravasation. However, the use of distal microparticle embolization in these small-caliber branches may have favored rapid vascular recanalization and contributed to early recurrent bleeding. Early rebleeding occurred 10 days later, raising concern for recurrent cecal bleeding despite prior embolization. Follow-up CTA did not reveal definite vascular lesions in the previously embolized cecal region (Figure 5), acting as a negative imaging landmark that supported the decision to pursue definitive surgical management.

**Figure 6:**
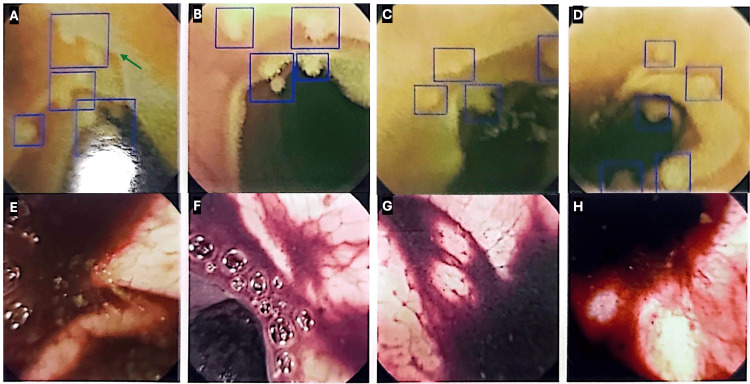
Small bowel capsule endoscopy localizing hemorrhage to the cecum. Capsule endoscopy images labeled A–H. A–D: Distal ileal views showing multiple non‑bleeding polypoid lesions without evidence of active small bowel bleeding; blue boxes indicate representative polypoid lesions, which were considered compatible with lymphoid hyperplasia and incidental to the severe hemoglobin drop. E–H: Cecal and colonic views demonstrating marked bleeding with persistent blood remnants visible at the end of the recording, localizing the hemorrhage to the right colon.

The patient continued to experience recurrent hematochezia and anemia despite endoscopic, pharmacologic, and endovascular therapy. Consequently, small bowel capsule endoscopy was performed late in the course to complete the small bowel assessment, showing normal jejunal mucosa and multiple non‑bleeding polypoid lesions in the distal ileum, without evidence of active small bowel bleeding. These distal ileal lesions were endoscopically compatible with lymphoid hyperplasia and were considered incidental findings, unlikely to be related to the life‑threatening hemoglobin drop to 5.2 g/dL. The capsule then traversed the ileocecal valve, where profuse cecal bleeding and residual blood throughout the colon were documented, further supporting a lower gastrointestinal source of bleeding (Figure 6).

Subsequently, given the persistence of hematochezia and anemia despite endoscopic, pharmacologic, endovascular, and capsule endoscopy findings indicating ongoing cecal bleeding, a multidisciplinary team decided to proceed with right hemicolectomy.

A detailed chronological summary of diagnostic and therapeutic interventions is provided in Table 1 (clinical timeline).

**Table 1:** Clinical timeline of diagnostic and therapeutic interventions.

Time point	Event / Intervention	Key findings	Clinical impact
Day 0	Presentation and admission	Persistent hematochezia with syncope and severe anemia were documented. Initial hemoglobin was 6.7 g/dL.	The presentation established clinically significant lower gastrointestinal bleeding and prompted urgent inpatient evaluation.
Hospital day 3	First colonoscopy	Colonoscopy showed right-sided diverticula and grade II hemorrhoids. No active bleeding was identified.	No definitive bleeding source was confirmed, so further evaluation remained necessary.
Hospital day 6	Repeat upper endoscopy and colonoscopy	Repeat bidirectional endoscopy again failed to identify a definite bleeding source.	Ongoing bleeding despite nondiagnostic endoscopy increased concern for an obscure source.
Hospital day 14	Third upper endoscopy with endoscopic therapy	Gastric erosions were treated endoscopically, but the findings did not fully explain the ongoing lower gastrointestinal bleeding.	Persistent bleeding after therapy prompted consideration of advanced imaging.
Hospital day 15	Dynamic CT angiography	Arterial-phase contrast extravasation and vascular ectasia were demonstrated in the cecum, consistent with angiodysplasia.	Imaging localized a probable bleeding source and guided endovascular treatment planning.
Hospital day 18	Selective arterial embolization with microparticles	Superselective transcatheter arterial embolization of the cecal branch was performed using microparticles.	Initial hemostasis was achieved after embolization.
Hospital day 28	Follow-up CT angiography	Follow-up CT angiography showed no definite vascular lesion.	Despite negative imaging, the patient remained transfusion-dependent, indicating persistent clinically relevant bleeding.
Hospital day 37	Capsule endoscopy	Capsule endoscopy revealed profuse cecal bleeding with residual blood throughout the colon.	The findings supported the cecum as the ongoing bleeding source and reinforced the need for definitive management.
Hospital day 43	Right hemicolectomy with ileostomy	Right hemicolectomy with ileostomy was performed.	Surgical management was undertaken for persistent cecal bleeding after prior diagnostic and endovascular interventions.

After surgery, the patient had an uneventful recovery and was discharged in stable condition. At six‑month follow‑up, he remained asymptomatic, with no further episodes of gastrointestinal bleeding, and his hemoglobin level had improved from 6.7 g/dL at presentation to 10.4 g/dL (reference range 13.7-17.5 g/dL for adult men), consistent with durable control of overt bleeding despite residual anemia.

From the patient’s perspective, the prolonged course of transfusion‑dependent bleeding, repeated hospitalizations, and multiple inconclusive endoscopic procedures was a major source of anxiety and fatigue. He expressed relief and satisfaction after the definitive surgery, reporting a marked improvement in energy levels and quality of life once the bleeding resolved and transfusions were no longer required.

## Discussion

Gastrointestinal angiodysplasia is a common vascular cause of recurrent or chronic lower gastrointestinal bleeding in older adults and is increasingly recognized in association with multiple comorbidities. Large population-based studies confirm that the prevalence of angiodysplasia rises with age and that conditions such as cirrhosis, cardiovascular disease, and chronic kidney disease significantly increase the risk of both lesion development and symptomatic bleeding [1-3]. In this context, our patient exhibited several risk factors, including advanced age, hypertension, and long-term nonsteroidal anti-inflammatory drug exposure. Chronic NSAID use not only predisposes to superficial mucosal injury but also amplifies bleeding from angiodysplastic lesions more than from structurally more robust sources such as peptic ulcers, by simultaneously impairing platelet aggregation and compromising primary hemostasis at fragile submucosal vessels. Recurrent NSAID-related microtrauma and ischemia-reperfusion cycles may sustain a local proangiogenic milieu that favors the formation, enlargement, and rapid re-recruitment of abnormal vascular tufts, helping to explain the refractory pattern of bleeding observed in our patient.

Endoscopic evaluation remains the cornerstone of diagnosis, but up to one-third of patients may have negative initial studies, particularly when lesions are located in the small bowel or when bleeding is intermittent [2,4-6]. In such cases, advanced imaging modalities such as capsule endoscopy and dynamic CTA have improved detection rates and facilitate localization of bleeding sites for targeted therapy [5-7]. Recent American College of Gastroenterology (ACG) guidelines emphasize a structured approach to acute lower gastrointestinal bleeding that integrates endoscopic, radiologic, and surgical modalities and highlights the need for individualized management in high-risk patients [8]. In our patient, repeated upper and lower endoscopies failed to identify an active source, whereas dynamic CTA demonstrated arterial-phase contrast extravasation and cecal vascular ectasia, supporting the diagnosis of angiodysplasia and guiding subsequent endovascular treatment.

Endoscopic therapies, including argon plasma coagulation, clipping, and novel submucosal coagulation techniques, achieve initial hemostasis in most cases but are limited by substantial long-term rebleeding rates, especially in multifocal or high-risk lesions [2,9-12]. In contrast, observational data suggest that, once the bleeding segment has been clearly localized, segmental colectomy offers more durable remission of lower gastrointestinal bleeding than repeat endoscopic or endovascular interventions in elderly patients with angiodysplasia, albeit at the cost of higher upfront procedural risk [1-3,13]. Transcatheter arterial embolization has emerged as an effective option for patients with ongoing or recurrent bleeding who are poor surgical candidates or have lesions in difficult locations, with reported technical success rates above 80% but clinically relevant rebleeding in a considerable proportion of cases [14-15]. Case reports specifically describing endovascular treatment of cecal angiodysplasia have highlighted both the feasibility of selective embolization and the risk of early rebleeding [16].

In the present case, early rebleeding 10 days after embolization was interpreted as recurrent cecal bleeding despite prior selective embolization, a scenario described in case series of lower gastrointestinal angiodysplasia, and likely reflected both technical and biological factors, including abundant collateral circulation, suspected early recanalization of distal embolized branches, and chronic NSAID‑associated proangiogenic stimuli. In this setting, chronic NSAID use may have acted as a persistent driver of microvascular fragility and neovascularization, contributing to the refractory pattern of bleeding despite technically successful embolization. Moreover, small, intermittent post‑embolization leaks may fall below the temporal and spatial resolution of CTA, as likely occurred in our patient, underscoring the limitations of follow‑up CTA alone to exclude ongoing low‑volume bleeding and the need to integrate imaging with the clinical course when deciding on definitive surgical management.

Intraoperatively, multiple diverticula with stigmata of bleeding were identified in the resected cecum and ascending colon. However, dynamic CTA had previously demonstrated arterial-phase contrast extravasation and vascular ectasia in the same segment, a pattern highly suggestive of angiodysplasia. The coexistence of diverticular disease and angiodysplasia in elderly patients with right-sided colonic bleeding has been described in pathological and clinical series, and both entities may share predisposing hemodynamic factors such as chronic venous congestion, increased wall tension, and age-related vascular degeneration [1-3,7,10]. In this setting, diverticula and angiodysplastic lesions should not be regarded as mutually exclusive diagnoses but rather as overlapping contributors within a spectrum of structural and vascular abnormalities in the right colon.

In patients with obscure gastrointestinal bleeding related to chronic NSAID or aspirin use, misoprostol has been shown to promote healing of small bowel ulcers and may improve mucosal outcomes, although its role in angiodysplasia-associated bleeding remains uncertain [17]. Our experience reinforces the concept that refractory angiodysplasia should be approached as a systemic vascular disorder requiring individualized, multimodal management within a multidisciplinary team. While repeated endoscopic and endovascular interventions are justified in many patients, clinicians must recognize scenarios in which these strategies are unlikely to provide durable control, and surgery should be considered earlier as a definitive option, particularly when the bleeding segment can be clearly localized, as in cecal disease [13-15].

In our case, repeat embolization would have required referral again to an external private interventional radiology center, and after experiencing recurrent bleeding and transfusion dependence despite prior minimally invasive therapies, the patient expressed a clear preference for a definitive surgical solution. Although repeat transcatheter embolization can control bleeding in a subset of patients with lower gastrointestinal angiodysplasia, observational series suggest that durable remission is more consistently achieved with segmental colectomy once the bleeding segment has been clearly localized, especially in elderly individuals with significant comorbidities. In selected patients such as ours, right hemicolectomy after failed embolization can secure durable control of overt bleeding and meaningful improvement in hemoglobin levels and overall clinical status [13-15].

From the patient’s perspective, the cumulative burden of recurrent hematochezia, repeated hospitalizations, and transfusion‑dependent anemia despite multiple minimally invasive procedures generated marked anxiety and fatigue. After discussing the option of seeking another embolization in the private sector versus definitive right hemicolectomy, he expressed a clear preference for surgery as a more durable solution. At follow‑up, he reported improved energy, greater confidence in daily activities, and relief at no longer needing further procedures or transfusions

Strengths and limitations

This case has several strengths, including detailed chronological documentation of multimodal diagnostics, the use of dynamic computed tomography angiography to precisely localize the source of cecal bleeding, and the integration of endoscopic, interventional, and surgical perspectives within a single clinical pathway. At the same time, it also highlights important limitations. First, the initial technical success of selective transcatheter arterial embolization may have created a false sense of security, potentially delaying consideration of definitive right hemicolectomy despite persistent transfusion‑dependent bleeding.

Second, from an interventional standpoint, embolization of cecal angiodysplasia is intrinsically challenging because of the rich collateral network of the ileocolic and right colic branches and the small caliber of the vasa recta, which increase the risk of incomplete penetration of embolic material and early rebleeding. In our case, distal embolization with microparticles achieved angiographic stasis but may have favored suspected early recanalization of the treated branches; more durable liquid embolic agents (such as cyanoacrylate glues or Onyx™ (Medtronic, Minneapolis, USA)) can provide deeper and more permanent occlusion but carry a higher risk of non‑target embolization and bowel ischemia in this vascular territory.

Finally, this report is limited by its single‑patient design and the absence of angiographic images suitable for publication, as well as the lack of a standardized algorithm to define the optimal threshold for transitioning from repeat endovascular therapy to surgery, underscoring the need for individualized multidisciplinary decision‑making in similar cases.

## Conclusions

Refractory lower gastrointestinal bleeding from a well‑localized cecal angiodysplasia that rapidly rebleeds after technically adequate embolization should be recognized as a warning sign that endovascular therapy is providing only transient hemostasis rather than definitive control. In this setting, dynamic computed tomography angiography is essential not only to confirm the bleeding site but also to delineate the segmental vascular anatomy, offering an anatomical roadmap to decide when early transition from repeated minimally invasive attempts to segmental colectomy is warranted.

Our case shows that, in elderly patients with right‑sided colonic bleeding and coexisting diverticular disease, promptly lowering the threshold for right hemicolectomy once rapid post‑embolization recurrence is documented can achieve durable hemostasis, improve hemoglobin levels, and meaningfully enhance recovery, and that in selected patients with well‑localized lesions who continue to bleed despite optimized endoscopic, pharmacologic, and endovascular therapy, segmental colectomy remains the necessary definitive treatment to secure durable hemostasis and meaningful clinical benefit.
